# RAGE signalling in obesity and diabetes: focus on the adipose tissue macrophage

**DOI:** 10.1080/21623945.2020.1817278

**Published:** 2020-09-06

**Authors:** Ziqian Feng, Luochen Zhu, Jianbo Wu

**Affiliations:** a Key Laboratory of Medical Electrophysiology of Ministry of Education, Collaborative Innovation Center for Prevention and Treatment of Cardiovascular Disease of Sichuan Province, Drug Discovery Research Center, Southwest Medical University, Luzhou, China; b Laboratory for Cardiovascular Pharmacology, Department of Pharmacology, School of Pharmacy, Southwest Medical University, Luzhou, Sichuan, China

**Keywords:** RAGE, white adipose tissue, macrophage, obesity, diabetes

## Abstract

The advanced glycosylation end product receptor (RAGE) acts as a recognition receptor and interacts with different types of ligands that form and accumulate in the tissues and circulation, such as diabetes, inflammation, insulin resistance, and obesity. In these environments, RAGE is expressed on the surface of various cells associated with tissue disturbance. This review mainly summarizes the characteristics of RAGE-related signalling, with a particular emphasis on the role of RAGE in the development of obesity. We also briefly describe the phenotypes and characteristics of macrophages and focus on the role of adipose tissue macrophages (ATMs) and the regulatory mechanisms in obesity, diabetes, and other related metabolic diseases. Besides, we will also elaborate on the prospect of new strategies for treating diabetes and obesity-related metabolic diseases by inhibiting RAGE signalling and regulating ATMs recruitment and polarization.

A large number of studies have shown that continuous high blood sugar in the body can cause the Maillard reaction of various proteins to form advanced glycation endproducts (AGEs). Elevated levels of AGEs can cause changes in the structure and function of extracellular proteins. So far, most studies on RAGE in monocytes/macrophages have explored the role of RAGE receptor and its related signal axis in type 2 diabetes, atherosclerosis, and insulin resistance.

## The biology of RAGE

The process of AGEs production is through non-enzymatic glycation of macromolecules with a reducing sugar, such as glucose or fructose. This accumulation can occur endogenously (hyperglycaemia, ageing, inflammation, renal failure, obesity and oxidative stress) or exogenously (high fat diet, processed food, chronic alcohol consumption and tobacco)[1]. The average body glycosylation reaction can proceed slowly. Once the body is ageing or continues to have high blood sugar, the glycosylation reaction quickly accelerates, thereby forming a large number of AGEs[1]. Among the various receptors of AGEs found at present, RAGE is a crucial receptor for AGEs to exert the primary mechanism of cells. RAGE is a new pattern recognition receptor and belongs to one of the members of the immunoglobulin superfamily. RAGE receptors are expressed on the surface of many cells, such as macrophages, mesangial cells, endothelial cells, etc [2]. which can combine with AGEs to form an AGE-RAGE axis to activate intracellular signalling pathways, thereby initiating a series of intracellular reactions. Experimental studies have shown that AGE-RAGE interaction can change cell signalling, promote gene expression, oxidative stress generation, and the release of proinflammatory molecules [3].

Under normal human conditions, the expression level of RAGE is very low; but when the cells in the body are in an activated or stressed state, the expression level of RAGE in the damaged cells is significantly increased. Therefore, RAGE plays an essential role in the pathological process of many diseases, such as diabetes, Alzheimer’s disease, vascular injury, and tumours. In addition, RAGE can recognize different ligands, and some endogenous ligands such as S100/calgranulins, high mobility group box-1 (HMGB1)[4], etc., they interact with RAGE after being released by damaged cells and activate some signalling pathways to increase tissue inflammation and damage [5]. In a hyperglycaemic environment, RAGE activation upregulates RAGE expression and leads to the translocation of the transcription factor (nuclear factor κ-light-chain-enhancer of activated B cells or NF-κB) into the nucleus. In addition, NF-κB inhibits GLo1 expression, inhibitor of AGE production, which leads to more AGEs production [6]. Diaphanous1 (DIAPH1) is a type of formin which is intracellular bind domain of RAGE. The interaction between DIAPH1 with RAGE is essential for RAGE ligand-mediated signalling in multiple cell types [7–9].

## RAGE and obese

Obesity is associated with higher risk of developing type 2 diabetes mellitus (T2DM) and cardiovascular disease (CVD). Beyond the management of the predisposing factors for the development of obesity, diabetes and CVD, there is a link between RAGE, inflammation, obesity, T2DM and CVD. RAGE expression is elevated in the adipose tissue of obese individuals, and it has been shown to have an effect on fat cell hypertrophy and insulin resistance in mice [10,11]. Masayo Monden and others showed that RAGE could directly regulate the process of fat cell hypertrophy in vitro. Inhibiting RAGE by siRNA intervention can significantly reduce fat cell hypertrophy. Furthermore, RAGE deficiency is related to the early suppression of toll-like receptor (Tlr) 2 mRNA expression in adipose tissue. Therefore, the regulation of Tlr2 by RAGE seems to be related to fat cell hypertrophy [7]. RAGE deficient mice fed on a high-fat diet are significantly lower in body weight, epididymal fat weight, and large size than WT mice, indicating that for high-fat diet-induced obesity, the RAGE knockout mice show apparent resistance [10]. More recently, HMGB1 was proposed to be an adipokine, and expressed 2-fold more in adipose tissue from obese individuals that acts as an innate pro-inflammatory mediator in WAT, which may be associated with insulin resistance and metabolic syndrome [1,12,13]. In the human body, the imbalance between energy intake and consumption will cause a series of diseases such as obesity. At this time, the body will cause some inflammatory cells to recruit to vital metabolic organs, primarily caused by the accumulation of macrophages into visceral fat tissue.

In metabolic diseases such as obesity and diabetes, the infiltration of macrophages and some immune cells can often be observed in adipose tissue, liver, and other parts, especially changes in macrophage M1 and M2 phenotypes regulate the overall occurrence of inflammatory reactions – process. More and more evidence shows that adipose tissue is not only an essential endocrine organ but also can secrete various biologically active substances such as leptin and adiponectin [14]. Among them, adipose tissue macrophages (ATMs) mainly maintain the homoeostasis and healthy function of adipose tissue by engulfing dead fat cells. Studies have shown that during obesity, adipose tissue will undergo metabolic inflammation, and ATMs will convert their phenotype to pro-inflammatory type, mainly manifested by increased infiltration of pro-inflammatory macrophages; once macrophages are activated, ATMs will produce some inflammatory factors interact with fat cells to increase the inflammatory response[15]. Therefore, it is possible to find new targets for treating obesity in macrophages, thereby reducing the metabolic syndrome associated with obesity.

Previous studies have shown that the expression of RAGE in adipose tissue of obese mice fed with a high-fat diet is elevated, and for RAGE-deficient mice fed with a high-fat diet, the adipose tissue is protected from adipocyte hypertrophy, macrophage infiltration, and insulin resistance. Therefore, some new treatment strategies can be found through ATMs or weakening RAGE signals [16].

## RAGE and ATMs

**Figure 1.**
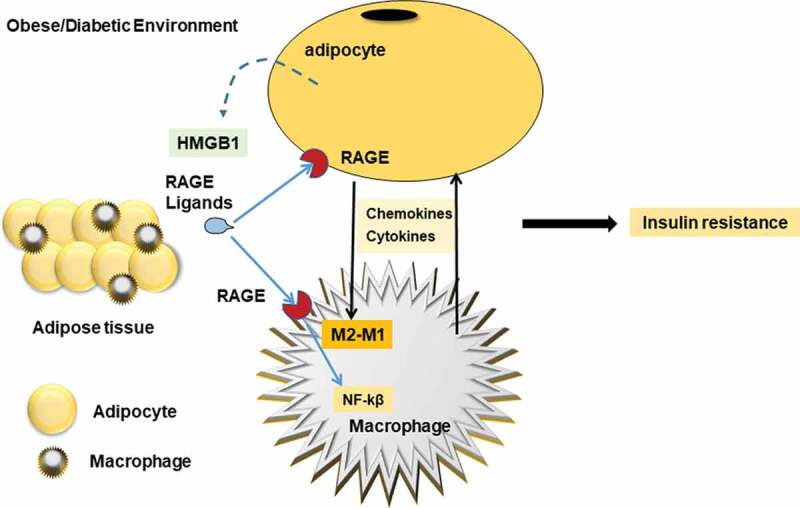
RAGE signalling in ATMs

ATMs are derived from circulating monocytes, but most of the evidence indicates that the infiltration of monocytes into adipose tissue is a complex phenomenon. As a multi-ligand receptor that recognizes inflammation signals, RAGE is highly expressed on monocytes and macrophages, and RAGE expression will be further enhanced when immune cells in the body are activated or damaged. Studies have shown that prevention of the interaction between RAGE and its ligands may be a therapeutic target for obesity and metabolic disorders [11]. Macrophages could increase the secretion of various cytokines and chemokines such as TNF-α, MCP-1, IL-6; Macrophages are further activated by these factors, which impairs the insulin signal in adipocytes and leads to systemic insulin resistance [17,18]. MCP-1 is secreted by hypertrophic adipocytes and stimulates the secretion of monocytes into macrophages by secreting pro-inflammatory cytokines. However, macrophages are considered to be ideal targets for the treatment of obesity and metabolic diseases. The potential treatment may be to suppress ATM-related inflammation, such as by reducing M1 pro-inflammatory macrophages or increasing M2 anti-inflammatory macrophages to alleviate Inflammation [19]. Earlier studies have found that macrophages in adipose tissue can promote the occurrence of obesity by inducing chronic inflammation [18], and can play a crucial role in insulin resistance caused by obesity. ATMs are mainly M2 anti-inflammatory macrophages under normal circumstances. With the development of obesity, M1 pro-inflammatory macrophages in adipose tissue gradually increase, and M2 anti-inflammatory macrophages gradually decrease, resulting in macrophages. The polarization state changed from M2 anti-inflammatory type to M1 pro-inflammatory type (Figure 1)[17]. It has also been reported in the literature before that RAGE ligand can induce macrophage activation, and it can also mediate monocyte/macrophage chemotaxis and upregulation of inflammatory factors [20,21].

RAGE by binding with ligands in both adipocytes and macrophages activates NF-κB signalling within WAT by influencing the release of cytokines and chemokine, which is related to insulin resistance.

The preliminary report of Gaens et al [22] . found that obese patients showed higher fasting blood glucose and fasting insulin levels than thin patients, but obese patients had lower glucose infusion rates than thin patients, so these obese patients showed very low Insulin sensitivity. However, they also further studied the expression of RAGE and RAGE ligand CML-AGE in adipose tissue by immunohistochemical staining. These staining results found that the accumulation of CML in subcutaneous adipose tissue of human obese subjects is more accessible to detect than that of lean adipose tissue and that the accumulation of CML in visceral adipose tissue of obese subjects is more than that of subcutaneous adipose tissue. Besides, they also confirmed in human adipose tissue that RAGE and CML-modified proteins are significantly expressed in adipocytes, CD68+ macrophages, and CD31-positive endothelial cells. The expression levels of CD163, IL-10, CD209d, Arg1, and CD209e in the visceral adipose tissue of Ager-/-mice fed a high-fat diet were significantly higher than those of wild-type mice [1], suggesting that RAGE can promote the development of obesity under the condition of high-fat diet feeding, and also promote the entry of F4/80-expressing macrophages into visceral fat. Although RAGE plays an essential role in obesity-induced by a high-fat diet, such as regulating body weight and adipose tissue macrophages, the exact mechanism of action is still unclear, and active research is needed to explore the role of RAGE in ATMs.

## Future research on the target therapeutics

There have been many studies in the past and present that explain the critical role of RAGE in the recruitment of monocytes/macrophages. Future research is necessary, and inhibition of RAGE may be an effective treatment [23]. It has also recently been reported that small molecule RAGE inhibitors can be used for research and clinical use [24]. At present, most of the animal models of these disease states, such as diabetes, cardiovascular disease, obesity, etc. have verified that RAGE as a therapeutic target can suppress chronic diseases, so in the future, we also hope to translate these meaningful findings into humans.
